# Vegetable Oil-Peroxidation Product ‘Hydroxynonenal’ Causes Hepatocyte Injury and Steatosis via Hsp70.1 and BHMT Disorders in the Monkey Liver

**DOI:** 10.3390/nu15081904

**Published:** 2023-04-14

**Authors:** Tetsumori Yamashima, Yurie Mori, Takuya Seike, Sharif Ahmed, Piyakarn Boontem, Shihui Li, Shinji Oikawa, Hatasu Kobayashi, Tatsuya Yamashita, Mitsuru Kikuchi, Shuichi Kaneko, Eishiro Mizukoshi

**Affiliations:** 1 Department of Psychiatry and Behavioral Science, Kanazawa University Graduate School of Medical Sciences, Kanazawa 920-8640, Japan; 2 Department of Gastroenterology, Kanazawa University Graduate School of Medical Sciences, Kanazawa 920-8640, Japan; 3 Department of Cell Metabolism and Nutrition, Kanazawa University Graduate School of Medical Sciences, Kanazawa 920-8640, Japan; 4 Department of Environmental and Molecular Medicine, Mie University Graduate School of Medicine, Tsu 514-8507, Japan

**Keywords:** betaine-homocysteine *S*-methyltransferase, *calpain-cathepsin hypothesis*, carbonylation, cell death, lysosomal rupture, phosphatidylcholine

## Abstract

Hsp70.1 has a dual function as a chaperone protein and lysosomal stabilizer. In 2009, we reported that calpain-mediated cleavage of carbonylated Hsp70.1 causes neuronal death by inducing lysosomal rupture in the hippocampal CA1 neurons of monkeys after transient brain ischemia. Recently, we also reported that consecutive injections of the vegetable oil-peroxidation product ‘hydroxynonenal’ induce hepatocyte death via a similar cascade in monkeys. As Hsp70.1 is also related to fatty acid β-oxidation in the liver, its deficiency causes fat accumulation. The genetic deletion of betaine-homocysteine *S*-methyltransferase (BHMT) was reported to perturb choline metabolism, inducing a decrease in phosphatidylcholine and resulting in hepatic steatosis. Here, focusing on Hsp70.1 and BHMT disorders, we studied the mechanisms of hepatocyte degeneration and steatosis. Monkey liver tissues with and without hydroxynonenal injections were compared using proteomics, immunoblotting, immunohistochemical, and electron microscopy-based analyses. Western blotting showed that neither Hsp70.1 nor BHMT were upregulated, but an increased cleavage was observed in both. Proteomics showed a marked downregulation of Hsp70.1, albeit a two-fold increase in the carbonylated BHMT. Hsp70.1 carbonylation was negligible, in contrast to the ischemic hippocampus, which was associated with ~10-fold increments. Although histologically, the control liver showed very little lipid deposition, numerous tiny lipid droplets were seen within and around the degenerating/dying hepatocytes in monkeys after the hydroxynonenal injections. Electron microscopy showed permeabilization/rupture of lysosomal membranes, dissolution of the mitochondria and rough ER membranes, and proliferation of abnormal peroxisomes. It is probable that the disruption of the rough ER caused impaired synthesis of the Hsp70.1 and BHMT proteins, while impairment of the mitochondria and peroxisomes contributed to the sustained generation of reactive oxygen species. In addition, hydroxynonenal-induced disorders facilitated degeneration and steatosis in the hepatocytes.

## 1. Introduction

Obesity and type 2 diabetes have reached epidemic proportions in Western countries [1]. Both conditions are closely associated with non-alcoholic fatty liver disease (NAFLD) and non-alcoholic steatohepatitis (NASH) [2,3,4,5]. The clinicopathological picture of both diseases resembles that of alcohol-induced liver injury; however, they occur in persons who consume little or no alcohol [6]. NAFLD is defined by excess fat accumulation and is classified into simple steatosis and NASH. NASH is a progressive subtype of NAFLD and was first defined by analogy to alcoholic hepatitis. NASH is characterized by the accumulation of fat in the liver (steatosis) along with inflammation and different degrees of scarring or fibrosis [7,8]. It is a potentially serious condition because approximately 10–25% of patients with NASH progress to liver cirrhosis and, eventually, to hepatocellular carcinoma [9,10]. The mechanism of progression from NAFLD to NASH is still not completely elucidated. Lipotoxicity is the harmful effect of lipid accumulation in non-adipose tissue, and currently, the occurrence of NASH is mainly viewed as a consequence of liver lipotoxicity. Using a range of experimental paradigms involving cultured cells, mice, monkeys, and humans, Seike et al. recently reported that hydroxynonenal can cause hepatocyte injury by inducing lysosomal membrane permeabilization/rupture [11].

It has been shown in the brain and pancreas that chronic exposure to hydroxynonenal induces the degeneration and death of brain neurons and Langerhans β cells via lysosomal membrane permeabilization/rupture [12,13,14]. However, the most important problem to be addressed is how hydroxynonenal induces lysosome-mediated cell degeneration and death. Lysosomal cell death has been widely accepted to occur in vivo, not only under pathological circumstances but also under physiological conditions. A perfect physiological example of lysosome-mediated programmed cell death is the post-lactational regression (involution) of the mammary gland, in which the alveolar mammary epithelium is removed and the gland is returned to its pre-pregnancy state. This is one of the most complex and highly regulated cell death programs to occur in the adult mammalian organism [15,16,17]. Milk fat globules formed during mammary gland involution upon the cessation of lactation are known to be toxic to epithelial cells. Perturbation of the lysosomal limiting membranes due to high concentrations of free fatty acids derived from milk triglycerides results in the controlled extra-lysosomal leakage of cathepsins, which culminates in the physiological cell death [17]. It is likely that certain lysosomal membrane proteins are vulnerable to excessive fatty acids. The same cascade may also occur in pathological conditions. As regards the mechanism of ischemic neuronal death, Yamashima et al. formulated the ‘*calpain-cathepsin hypothesis*’ in 1998 [18] and in 2009 modified it by adding the concept of the ‘calpain-mediated cleavage of carbonylated heat shock protein 70.1 (Hsp70.1)’ [19,20]. They reported that Hsp70.1 (also called Hsp70 or Hsp72), which has a dual function as a molecular chaperone and lysosomal stabilizer, is vulnerable to cleavage by activated μ-calpain, especially after carbonylation by hydroxynonenal. After the specific oxidative modification that is followed by μ-calpain-mediated cleavage, functional Hsp70.1 decreases, which result in the accumulation of garbage proteins and/or damaged organelles and the permeabilization/rupture of the lysosomal membrane. Although best known for its chaperone functions, Hsp70.1 also has a widely accepted role in the regulation of metabolism. However, very little is known about the role of Hsp70.1 in liver metabolism [21,22].

The liver is the major site of choline metabolism, where it is found primarily as phosphatidylcholine [23]. In normal hepatocytes, a critical function of lipid droplet formation is the regulation of the intracellular concentrations of unesterified fatty acids and cholesterol, because they are cytotoxic at increased concentrations [24]. Phosphatidylcholine in the lipid droplet monolayer acts as a surfactant to prevent the coalescing of each lipid droplet, as this process would yield larger lipid droplets which are less likely to undergo lipolysis [25]. Hepatic steatosis develops when fatty acid influx, de novo hepatic lipogenesis, or triglyceride (triacylglycerol) synthesis exceeds lipid efflux or oxidation. Alterations in choline and phosphatidylcholine metabolism has an impact on hepatic steatosis [26]. In the non-fatty liver, there is a homeostatic balance between triglyceride synthesis and efflux. Given that phosphatidylcholine is required for the efflux of very low-density lipoprotein (VLDL), a reduction in phosphatidylcholine would cause triglyceride accumulation in the NAFLD liver [27].

The mechanisms that alter susceptibility to hepatic steatosis are poorly understood in both NAFLD and NASH. Betaine-homocysteine *S*-methyltransferase (BHMT: EC2.1.1.5) is an enzyme that is predominantly found in the liver as a major regulator of choline metabolism [28,29]. However, aside from its role in ‘one carbon metabolism’, the mechanisms of BHMT function and disorder are incompletely understood. It catalyzes the formation of methionine from homocysteine, using the choline metabolite betaine as a methyl donor. Therefore, BHMT deficiency leads to elevated betaine and homocysteine concentrations and a reduced choline concentration [30]. Consequently, choline deficiency influences hepatic lipid accumulation by reducing the phosphatidylcholine concentration. BHMT overexpression increases phosphatidylcholine synthesis, leading to reduced hepatic lipid accumulation [31], whereas BHMT deficiency leads to fatty liver [30]. Protein oxidation is currently thought to be an important factor in various pathological conditions such as Alzheimer’s and Parkinson’s diseases, cardiovascular disease, type 2 diabetes, and stroke (ischemia/reperfusion injury) [32]. The most widely studied product of protein oxidation is the formation of carbonyl derivatives on the side chains of certain amino acids, e.g., arginine, lysine, and proline [33,34,35]. For example, via a proteomics analysis of ischemic neuronal death, Oikawa et al. found more than a 10-fold increase in carbonylated Hsp70.1, which was followed by calpain-mediated cleavage [35]. Accordingly, in this study, we specifically focused on the protein carbonylation of both Hsp70.1 and BHMT in our proteomics (2D-Oxyblot) analysis because carbonylated BHMT was previously found in a rodent model of alcoholic steatosis and aging liver [32,36].

In order to better understand the mechanism of hepatic injury and steatosis, we conducted a current study on normal chow-fed, young monkeys after consecutive injections of synthetic hydroxynonenal. Therein, hydroxynonenal was demonstrated to be an instigator of hepatic injury, impairing the Hsp70.1 and BHMT proteins and disturbing lysosomal structure. By focusing on disorders of both Hsp70.1 and BHMT, the mechanisms of hepatocyte degeneration/death and steatosis are discussed.

## 2. Materials and Methods

### 2.1. Animal Experiments

After approval from the ethics and animal welfare committee regarding animal experimentation, seven young (aged 4–5 years old; expected life span: 25–35 years) Japanese macaque monkeys (*Macaca fuscata*) were supplied by the National Bio-Resource Project (NBRP) for this study. These monkeys were reared in a large cage with autofeeding and autocleaning machines and appropriate toys for 1 year to facilitate acclimation. The room temperature was maintained at 22–24 °C with a humidity of 40–50%. They were fed approximately 100 g × 2/day of a non-purified diet (CLEA Old World Monkey Diet CMK-2, containing 344.7 kcal/100 g but only 4.05% crude fat, CLEA Japan, Inc., Tokyo, Japan). Apples, bananas, or sweet potatoes were given twice per week. Animal care staff monitored the health and well-being of the animals by checking their consumption of foods, their pupilar reflex to light, and their standing and jumping behavior. 

When 5 mg of hydoxynonenal (Cayman Chemical, Ann Arbor, MI, USA) was administered to the monkeys, the serum hydoxynonenal concentration immediately after the intravenous injection was estimated to be 60 μM/L, as calculated from the blood volume converted from the monkey’s body weight of 6–7 Kg. On the basis of a report that the serum hydroxynonenal concentration in the patients with Alzheimer’s disease is 20.65 μM/L at the median level (range: 6.02–25.20) [37] and considering the fact that hydoxynonenal is rapidly metabolized in the liver, we assumed that the concentration of hydoxynonenal in the monkey serum after the 5 mg/week injection was similar to that of a pathological state in humans. Therefore, seven monkeys were randomly divided into sham-operated controls (n = 3) and those receiving hydoxynonenal injections (n = 4). In the latter group, 5 mg of hydroxynonenal was intravenously injected every week for 24 weeks (a total amount of 120 mg for each monkey).

All experimental procedures were in accordance with the guidelines for the Care and Use of Laboratory Animals of the National Institutes of Health, which met the ‘International Guiding Principles for Biomedical Research Involving Animals’, as issued by the Council for the International Organizations of Medical Sciences. The protocol was approved by the Committee on the Ethics of Animal Experiments of the Kanazawa University Graduate School of Medical Sciences (Protocol Number: AP-153613, 194062). 

### 2.2. Blood Sampling

In the 4 monkeys receiving hydroxynonenal injections, venous blood was sampled once every month for 5 months prior to the hydroxynonenal injections (the total sample number = 20) and for 6 months during the injection periods (the total sample number = 24). Thus, blood data were recorded for 11 months in all 4 animals. Blood sampling was also performed on the 3 control monkeys. To establish when liver injuries had occurred in the hydroxynonenal-treated monkeys, the average level at each time point over the 11 months was considered. Blood samples were taken at the time of each hydroxynonenal injection, but the first blood sample was taken the week following the initial injection. A two-way ANOVA with Bonferroni’s post-test was used to compare the data before and after hydroxynonenal injections, and *p* < 0.05 was considered significant.

### 2.3. Tissue Collection

Monkeys after hydroxynonenal injections (n = 4) and sham-operated controls (n = 3) were immobilized via an intramuscular injection of 10 mg/Kg body weight ketamine hydrochloride. In addition, to ameliorate animal suffering, the monkey was deeply anesthetized with 1–1.5% halothane plus 60% nitrous oxide. After the perfusion of 500 mL of saline through the left ventricle, the liver and pancreas were removed. Half of the tissue was fixed in 4% paraformaldehyde for light microscopy and 2.5% glutaraldehyde for electron microscopy. The remaining half was stored at −80 °C in a deep freezer for phosphatidylcholine concentration measurements, Western blotting, and proteomics analyses.

### 2.4. Analysis of Phosphatidylcholine Concentration in the Liver Tissues

The liver tissues with (n = 3) and without (n = 3) hydroxynonenal injections were used for the measurement of the phosphatidylcholine concentration. Phosphatidylcholine analysis was conducted according to the Lipidome Lab Phospholipid Enzymatic Fluorometric Assays package (Lipidome Lab, Akita, Japan). First, total lipids were extracted from 100 mg of monkey liver samples using the Bligh–Dyer method [38]. An aliquot of the lower/organic phase was evaporated to dryness under N_2_ gas, and the residue was dissolved in methanol for the phosphatidylcholine measurement. Details of the enzymatic fluorometric assays to quantify phospholipid classes were previously described [39]. Briefly, each sample (2.5 mg tissue/mL) was added to Reagent C1 (100 U/mL GPL-PLD, 1.5 mM CaCl_2_, 50 mM NaCl, and 50 mM Tris-HCl, pH 7.4) and incubated at 37 °C for 30 min. After incubation, Reagent C2 (4 U/mL choline oxidase, 5 U/mL peroxidase, 300 μM Amplex Red, 0.2% Triton X-100, 50 mM NaCl, and 50 mM Tris-HCl, pH 7.4) was added to each well and incubated at room temperature for 30 min. Then, Amplex Red Stop Reagent was added. The fluorescence intensity was measured at excitation at 544 nm and emission at 590 nm using a microplate reader (SpectraMax iD3, Molecular Device, San Jose, CA, USA).

### 2.5. Western Blotting

For Western blotting of the liver tissues after the hydroxynonenal injections, the pancreas tissue was utilized as a positive control of the stress-induced upregulation of Hsp70.1 [14]. Total protein extraction was performed for each sample using a protease inhibitor cocktail (Sigma-Aldrich, St. Louis, MO, USA) and PhosSTOP phosphatase inhibitor cocktail tablets (Roche, Munich, Germany). After centrifugation at 12,000 rpm for 10 min, the supernatant proteins were determined using the Bradford assay (Thermo Fisher, Waltham, MA, USA). In total, 20 μg of proteins were separated using SDS-PAGE in SuperSep (TM) Ace 5–20% gel (Wako, Wako, Japan) at 40 mA for 1 h. The total proteins were transferred to a PVDF membrane (Millipore, Burlington, MA, USA). The transferred protein quantities were calculated with Ponseau S solution (Sigma-Aldrich, USA). The transferred proteins were blocked with 1% bovine serum albumin (KPL DetectorTM Block, USA) for 1 h. 

The blots were incubated with mouse monoclonal anti-human Hsp70 antibody (BD Bioscience, San Jose, CA, USA) at a dilution of 1:4000, rabbit anti-human BHTM antibody (abcam, USA) at 1:1000, or mouse anti-human hydroxynonenal antibody (JaICA, Tokyo, Japan) at 1:500 overnight. GAPDH or β-actin were utilized as an internal control (Sigma-Aldrich, USA) at a dilution of 10,000. The immunoblots were subsequently incubated for 1 h with secondary antibodies of anti-mouse IgG (Santa Cruz, Santa Cruz, CA, USA) at a dilution of 1:10,000 or anti-rabbit IgG (Sigma, USA) at a dilution of 1:10,000. An enhanced chemiluminescence (ECL) HRP substrate detection kit (Millipore, USA) was used to visualize the reactive protein bands with the ImageQuant LAS 4000 mini (GE Life Science, Issaquah, WA, USA).

### 2.6. Detection of Carbonyl-Modified Proteins (2D-Oxyblot Analysis)

The liver tissues were directly transferred into a reaction tube containing 100 µL of lysis buffer (30 mM Tris-HCl, 7 M urea, 2 M thiourea, 4 % *w*/*v* 3-[(3-cholamidopropyl) dimethylammonio] propanesulfonate, a protease inhibitor cocktail, pH 8.5). The hippocampus after transient ischemia was utilized as a positive control of Hsp70.1 carbonylation [35]. The tissue samples were homogenized using the Sample Grinding Kit (GE Healthcare UK Ltd., Amersham, England) and incubated on ice for 60 min. All samples were centrifuged at 30,000× *g* for 30 min at 4 °C. The supernatant was collected and stored at −80 °C. The total protein of the sample was quantified using the Bradford assay, and each sample was prepared to contain 100 µg of protein. Derivatization with 2,4-dinitrophenylhydrazine (DNPH) was conducted according to the procedure of Nakamura and Goto [40]. Carbonylated proteins in each protein sample (100 µg of protein) were labeled by derivatization of the carbonyl group with 2,4-dinitrophenylhydrazone (DNP) via a reaction with DNPH and separated by two-dimensional electrophoresis. Two-dimensional electrophoresis and the subsequent immunoblotting for protein carbonyls were performed as previously described [41]. For the first dimension, isoelectric focusing was performed on IPG strips (IPG, pH 3–10 NL strips, 24 cm, GE Healthcare) and an Ettan IPGphor isoelectric focusing system (GE Healthcare). For the second dimension, 12.5% SDS–polyacrylamide gel electrophoresis was performed using an Ettan DALT Six large electrophoresis system (GE Healthcare). 

After the second dimension, the proteins from the gels were transferred to a polyvinylidene fluoride membrane (Immobilon-P transfer membrane; Millipore), using a TE77 semi-dry transfer unit (GE Healthcare) at 50 V for 30 min. The DNP adduct of the carbonyls of the proteins was detected on the PVDF membrane using an immunoblot kit (OxySelect^TM^ Protein Carbonyl Immunoblot Kit, CELL BIOLABS, INC., San Diego, CA, USA). The chemiluminescence signal was detected on X-ray films. The spot intensities of the carbonylated proteins were quantified using PDQuest ver. 8.0 (Bio-Rad, Hercules, CA, USA). We repeatedly confirmed the reproducibility of the 2D-Oxyblot analysis. The specific oxidation was estimated as the relative carbonyl level (obtained from 2D-Oxyblot) corresponding to relative protein expression (obtained from 2D-DIGE). For protein identification, spots were excised from the 2D gels obtained with non-DNPH-treated samples and analyzed using mass spectrometry. Mass analysis was performed using a matrix-assisted laser desorption ionization time-of-flight tandem mass spectrometer (MALDI-TOF/TOF MS; 4800 Plus MALDI-TOF/TOF^TM^ Analyzer, AB SCIEX, Framingham, MA, USA). Protein identification was performed using the MS/MS ion search tool in ProteinPilot software version 4.0 (AB SCIEX, Framingham, MA, USA).

### 2.7. Two-Dimensional Differential in Gel Electrophoretic (2D-DIGE) Analysis

Each soluble protein sample (25 µg of protein) was minimally labeled with CyDye DIGE fluors according to the manufacturer’s protocol (GE Healthcare). Each Cy3-labeled sample was combined with an equal amount of a Cy5-labeled sample, and the pooled Cy2-labeled sample was added as an internal standard. After incubation on ice for 30 min in the dark, the samples were separated using 2D electrophoresis [42]. The 2D-DIGE gels were scanned on a Typhoon FLA 9500 (GE Healthcare). Spot detection, gel matching, and statistical analysis were performed with the DeCyder 2D software version 7.2 (GE Healthcare). 

As a result of (1) the heterogeneity of hydroxynonenal-induced hepatic lesions in the given monkey and (2) the small number of experimental animals because of the high costs of both Japan *macaque* monkeys and the synthetic hydroxynonenal, statistical analyses of the phosphatidylcholine measurements, Western blotting results, and proteomics data were not conducted.

### 2.8. Histological and Immunofluorescence Histochemical Analyses

After fixation with 4% paraformaldehyde for 2 weeks, the liver tissues were embedded in paraffin, and 5 μm sections were stained with hematoxylin-eosin (H-E). For the immunofluorescence histochemistry, the cryoprotected liver tissues embedded in the OCT medium (Sakura Finetek, Osaka, Japan) were cut using a cryotome (Tissue-Tek1Polar1, Sakura, Japan), and 5 μm sections were immersed in heated 0.01% citrate retrieval buffer to induce epitope retrieval. Non-specific staining was blocked with 1% bovine serum albumin (Nacalai tesque, Kyoto, Japan), and samples were incubated overnight at 4 °C with the primary antibodies at a dilution of 1:100. We used mouse monoclonal anti-human Hsp70 (BD Bioscience, USA), rabbit anti-human activated μ-calpain (only recognizing the activated form of 76 kDa, order-made from the Peptide Institute, Ibaraki, Japan), and rabbit anti-human cathepsin B (Cell Signaling, USA) antibodies. After washing, the sections were incubated for 30 min with secondary antibodies; Alexa FluorTM 594 goat anti-mouse IgG [H+L] (Invitrogen, Waltham, MA, USA) or Alexa FluorTM 488 goat anti-rabbit IgG (Invitrogen, USA) at a dilution of 1:500. To block autofluorescent staining, the Autofluorescence Quenching Kit (Vector Laboratories, Newark, CA, USA) was utilized. The immunoreactivity was observed with a laser confocal microscope (LSM5 PASCAL, Software ZEN 2009, Carl Zeiss, Germany).

### 2.9. Ultrastructural Analysis

For the electron microscopic analysis, small specimens of liver tissue were fixed with 2.5% glutaraldehyde for 2 h and 1% OsO4 for 1 h. Subsequently, they were dehydrated with graded acetone and embedded in resin (Quetol 812, Nisshin EM Co., Tokyo, Japan). Then, thin sections were produced for staining with 0.5% toluidine blue (T-B). After trimming the resin-embedded tissues, the ultrathin (70 nm) sections of the selected area were stained with uranyl acetate (15 min) and lead citrate (3 min) and observed using an electron microscope (JEM-1400 Plus, JEOL Ltd., Tokyo, Japan).

## 3. Results

**Figure 1:**
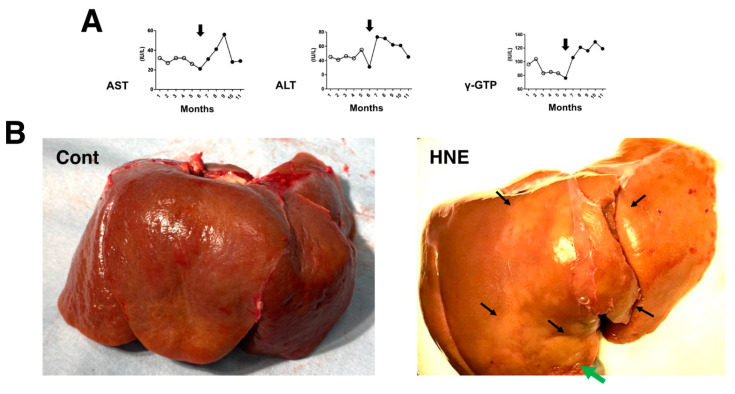
Blood data (**A**) and inspection of the liver at autopsy (**B**). During the 5 mg/week hydroxynonenal (HNE) injections (total dose: 120 mg), the blood analysis showed increased levels of AST, ALT, and γ-GTP ((**A**), closed circles), compared to the controls prior to hydroxynonenal injections ((**A**), open circles). The surface of the control liver looked diffusely reddish-brown at autopsy ((**B**), Cont), while at autopsy 6 months after the initial hydroxynonenal injections, the liver surface of the hydroxynonenal-treated monkeys exhibited heterogeneous, whitish-yellow discoloration ((**B**), HNE, black and green arrows) intermingled with dark-brown areas. These whitish-yellow areas histologically exhibited severe degeneration, while the dark-brown area exhibited mild degeneration.

**Figure 2:**
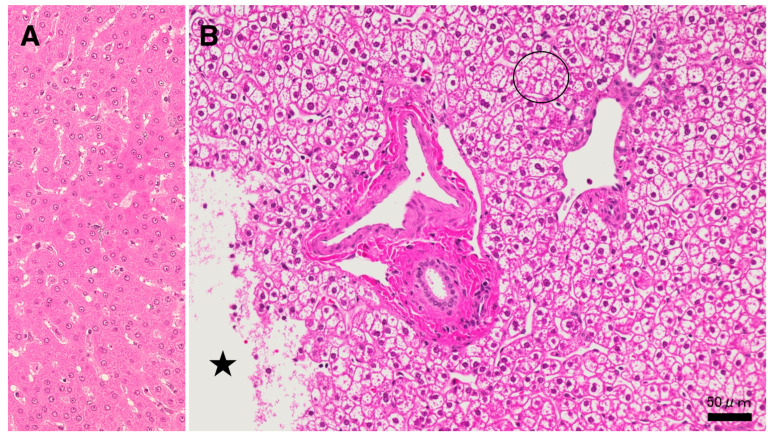
Histological findings from the hydroxynonenal-treated liver. In light microscopy, the whitish-yellow portion (Figure 1B, HNE) of the liver after the consecutive hydroxynonenal injections exhibited widespread fatty degeneration of hepatocytes and focal necrosis ((**B**), star). The cytoplasm of the affected hepatocytes around the portal triad ((**B**), circle) was translucent, presumably due to the dissolution of lipid components during ethanol dehydration. This was in marked contrast to the normal hepatocyte (**A**). Star: focal necrotic area, H-E staining.

**Figure 3:**
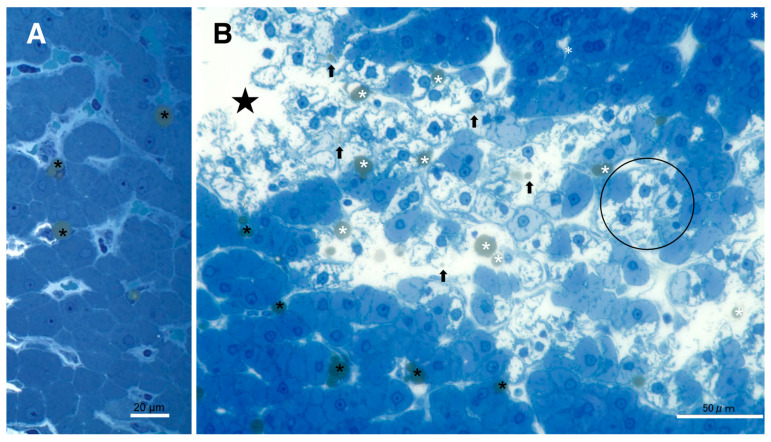
Accumulation of lipid droplets after the hydroxynonenal injections. In the control liver (**A**), lipid droplets were mainly seen within Ito cells next to the sinusoid lumen ((**A**), black asterisks). In contrast, after hydroxynonenal injections, many lipid droplets were seen within and outside the degenerating/necrotic hepatocytes ((**B**); white asterisks and arrows). The double fixation for the electron microscopic preparation preserved lipid components against acetone dehydration. The translucent cytoplasm of degenerating hepatocytes ((**B**), circle) exhibited dissolution of cytoplasmic organelles, as confirmed by electron microscopy (Figure 10B, circle). Star: focal necrotic area, T-B staining.

The comparison of blood data before (n = 20 collected for 5 months; Figure 1A, open circles) and after (n = 24 collected for 6 months; Figure 1A, closed circles) the start (Figure 1A, arrows) of hydroxynonenal injections showed a significant (*p* < 0.05) increase in aspartate aminotransferase (AST), alanine aminotransferase (ALT), and γ-glutamyl transferase (γ-GTP). This was observed for 24 weeks in all four of the monkeys that received 5 mg/week (a total dose of 120 mg for each monkey) hydroxynonenal injections. The difference was almost the same when compared with the blood data of the three control monkeys that did not receive hydroxynonenal injections. Because the increases in AST, ALT, and γ-GPT became pronounced in the second month after the start of the 5 mg/week × 4 injections, i.e., after four injections in the four monkeys, hepatic injury was thought to occur within a couple of weeks of the initial injection (Figure 1A, arrows). At autopsy 6 months later, the surface of the liver of the non-injected control monkeys looked reddish-brown (Figure 1B, Cont), whereas the surface of the hydroxynonenal-treated liver exhibited a heterogenous, whitish-yellow discoloration (Figure 1B, HNE, arrows) intermingled with dark-brown areas. The whitish-yellow portion histologically exhibited severe hepatocyte degeneration with the accumulation of lipid droplets (Figure 2B and Figure 3B). In contrast, the surrounding dark-brown area showed mild degeneration. 

The concentration of phosphatidylcholine in the liver tissues of the three control monkeys was 19.39, 18.17, and 19.74 (average: 19.10) μg/mg tissue, while that of the three hydroxynonenal-treated monkeys was 22.26, 17.46, and 14.85 (average: 18.19) μg/mg tissue. After hydroxynonenal injections, one monkey exhibited a higher phosphatidylcholine level, and two monkeys exhibited a lower phosphatidylcholine level compared to the controls. By considering the heterogeneity of hepatic legions in the monkeys (Figure 1B, HNE), we speculated that phosphatidylcholine concentration also exhibited a regional heterogeneity. Interestingly, the monkey with the lowest phosphatidylcholine concentration (14.85 μg/mg tissue) had the most severe hepatic injury among the three monkeys studied.

Since lysosomal cell death was thought to occur in the liver in the same manner as reported in the ischemic brain [19,20], the following light microscopic, immunofluorescence histochemical, and electron microscopic analyses were focused on the lysosomal membrane integrity of the affected hepatocytes. In the microscopic observation, the whitish-yellow area exhibited widespread foamy degeneration, which was comprised of hepatocytes with advanced cytoplasmic degeneration around the portal triad (Figure 2B). The translucent cytoplasm indicated the almost complete dissolution of lipid components during ethanol dehydration for the microscopic preparation. Many tiny granules were seen in the cytoplasm (Figure 2B, circle). These were thought to be degenerating mitochondria and peroxisomes, which was confirmed in the electron microscopy (Figures 8A,B and 9B,C). A focal necrotic area was detected (Figure 2B, star; Figure 3B, star), but apoptotic bodies were not observed. The necrotic area had been infiltrated by inflammatory cells. The degenerating hepatocytes (Figure 2B) did not show ballooning and were as large as normal hepatocytes (Figure 2A). The cell boundary was distinct, which was compatible with a thickened basement membrane, as shown in the ultrastructural observation (Figure 8A, BM). In the degenerating hepatocytes, the nuclei often showed a dense chromatin region compared to the controls. These histological changes were not observed in the control livers, the cytoplasm of which was filled with an eosinophilic substance (Figure 2A). The degenerating hepatocytes were markedly different from the control hepatocytes.

The procedures used for the electron microscopic observations involved preserving lipid components within the tissues via double fixations using glutaraldehyde and OsO_4_. Accordingly, T-B staining of the semi-thin sections of resin-embedded tissues revealed slightly yellowish lipid droplets in both the control (Figure 3A) and the hydroxynonenal-treated degenerating (Figure 3B) hepatocytes. In the control liver, lipid droplets were mainly seen within Ito cells, which were localized in the subendothelial space of the sinusoids (Figure 3A, black asterisks). The hepatocytes, if present, exhibited tiny lipid droplets. In contrast, in the livers after the hydroxynonenal injections, other than in the Ito cells (Figure 3B, black asterisks), many small lipid droplets were seen within and in the vicinity of degenerating/dying hepatocytes (Figure 3B, white asterisks, arrows). Lipid droplets measuring 2–15 μm were formed within the degenerating and dying hepatocytes, the cytoplasm of which was translucent in the light microscopic observation (Figure 2B and Figure 3B, circle) or exhibited a decreased electron density in the electron microscopic observation (Figure 10B, circle). Abundant cell organelle debris appeared as coarse granules within and around the dead cells. The hepatocytes with and without hydroxynonenal injections contrasted remarkably in both the H-E and T-B staining (Figure 2 and Figure 3). 

**Figure 4:**
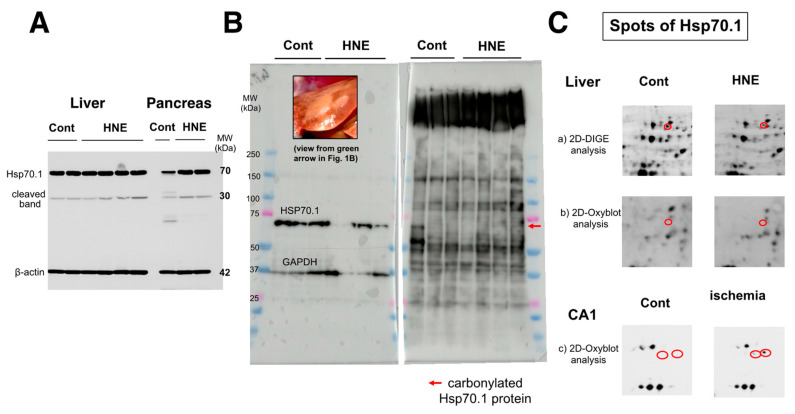
Western blotting (**A**,**B**,**left**), SDS-PAGE (**B**,**right**), and proteomics analyses (**C**) of Hsp70.1. Via Western blotting after electrophoresis on the same gel, in both the liver (dark-brown portion) and pancreas, Hsp70.1 cleavage was increased after hydroxynonenal injections (**A**, HNE), compared to the control (**A**, Cont). Despite being a stress-inducible protein, Hsp70.1 exhibited no upregulation of the naïve protein as a result of a long-term insult via consecutive hydroxynonenal injections in the liver (**A**, liver). This was in marked contrast to the pancreas, which exhibited upregulation of Hsp70.1 naïve proteins after the same insult (**A**, pancreas). Western blotting using the whitish-yellow portion showed downregulation of Hsp70.1 after hydroxynonenal injections (**B**, left). SDS-PAGE using the whitish-yellow portion (Figure 1B HNE, green arrow; **B**, left, upper) and anti-DNP antibody showed the negligible expression of carbonylated Hsp70.1 (**B**, HNE). These were consistent with the data from the 2D-Oxyblot analysis using the anti-DNP antibody, which showed the negligible expression of carbonylated Hsp70.1 (**C-b**), although 2D-DIGE showed the decreased expression of Hsp70.1 after the hydroxynonenal injections (**C-a**). The reduction in carbonylated Hsp70.1 after consecutive hydroxynonenal injections was in marked contrast to the significant increase in carbonylated Hsp70.1 in the hippocampal CA1 after transient ischemia (**C-c**) [35].

As already demonstrated in brain tissue [20], calpain-mediated cleavage of Hsp70.1 was also shown to be increased in the dark-brown portion of the liver, compared to the controls (Figure 4A, Liver). However, surprisingly, upregulation of Hsp70.1, albeit a stress-inducible protein, was not observed in response to the chronic insult of hydroxynonenal injections. This was in marked contrast to the pancreas of the same monkey, which exhibited increases in both Hsp70.1′s main band and its cleavage during simultaneous electrophoresis (Figure 4A, pancreas). Using the whitish-yellow portion (Figure 1B, green arrow; Figure 4B, upper rectangle) of the liver associated with severe degeneration, Western blotting showed the downregulation of Hsp70.1 after the hydroxynonenal injections (Figure 4B, left). In addition, SDS-PAGE followed by immunostaining using the anti-DNP antibody showed increases in various adduct proteins, which were modified by hydroxynonenal. However, the band indicating carbonylated Hsp70.1 was negligible at around 70 kDa (Figure 4B, right, red arrow). 

The 2D-Oxyblot analysis using the anti-DNP antibody also showed the negligible expression of carbonylated Hsp70.1 (Figure 4C-b), although the 2D-DIGE analysis confirmed the expression of the Hsp70.1 protein (Figure 4C-a). In addition, the 2D-DIGE analysis showed the downregulation of the Hsp70.1 protein on the corresponding spot. In our study, using the same proteomics procedure, the 2D-Oxyblot analysis of the hippocampus (CA1) of the monkey brains showed a significant upregulation of carbonylated Hsp70.1 after transient ischemia, as shown in (Figure 4C-c) [35]. In a previous study, the insult to the brain was merely an acute transient ischemic insult, and the analysis was performed 3–7 days after the insult. In contrast, the insult to the liver in the present experiment resulted from chronic exposure to hydroxynonenal for 24 weeks, and the analysis was performed up to 6 months after the first hydroxynonenal injection. Therefore, it is likely that due to both (1) impairments in synthesis related to rough ER damages as demonstrated by electron microscopy (Figure 8), and (2) the long-term cleavage of carbonylated Hsp70.1 (Figure 4A), the reduction in both naïve and carbonylated Hsp70.1 proteins (Figure 4C-a,C-b) progressed step by step for the 24 weeks of hydroxynonenal injections. 

**Figure 5:**
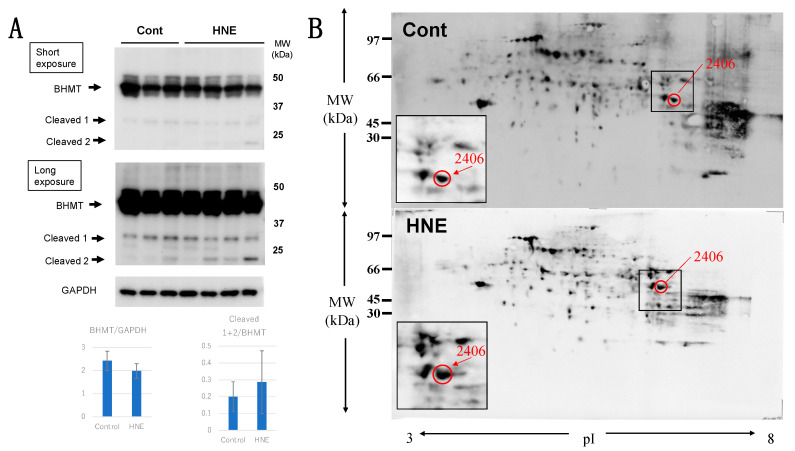
Western blotting (**A**) and 2D-Oxyblot analyses (**B**) of BHMT in the control and hydroxynonenal (HNE)-treated livers. Western blotting using the anti-BHMT antibody showed both a decrease in the BHMT naïve proteins ((**A**), BHMT) and an approximate 1.5-fold increase in BHMT cleaved bands after both short and long exposures ((**A**), cleaved 1 and 2), compared to the controls. As BHMT carbonylation was previously reported in the rodent model of alcoholic steatosis [36], here we focused on spots with similar MW and pI values in the 2D-Oxyblot analysis. Spot 2406 with a pI of 6.58 and MW of 44,998 (red circles) was identified as BHMT in the mass spectrometry analysis and via database matching. Compared to the controls ((**B**), Cont), the degenerating liver after hydroxynonenal injections ((**B**), HNE) showed a two-fold increase in the specific oxidation of BHMT ((**A**,**B**) rectangles). In contrast, spots of carbonylated Hsp70.1 were not detected in the 2D-Oxyblot analysis (Figure 4C-b), which was in marked contrast to the ischemic brain [35] (Figure 4C-c). As the 2D electrophoresis gel is much larger than that of SDS-PAGE, the molecular marker only shows approximate positions.

In the Western blotting analysis using the anti-BHMT antibody, the liver tissues after hydroxynonenal injections showed both a decrease in the BHMT naïve protein and a 1.5-fold increase in BHMT cleaved bands after both short and long exposures, compared to the controls (Figure 5A). The carbonylation of BHMT was previously reported in a rodent model of alcoholic steatosis to be a target protein of fatty liver development [36]. Accordingly, in the 2D-Oxyblot analysis, we focused on spots with similar MW and pI values as BHMT. The 2406 spots, with a pI of 6.58 and MW of 44,998, were identified as BHMT in the mass spectrometry analysis and via database matching (Figure 5B). The 2406 spots (Figure 5B, red circles) in both liver tissues with (HNE) and without (Cont) hydroxynonenal injections were enlarged (Figure 5B, rectangles). An approximately two-fold increase in carbonylated BHMT was observed in the densitometric analysis. 

**Figure 6:**
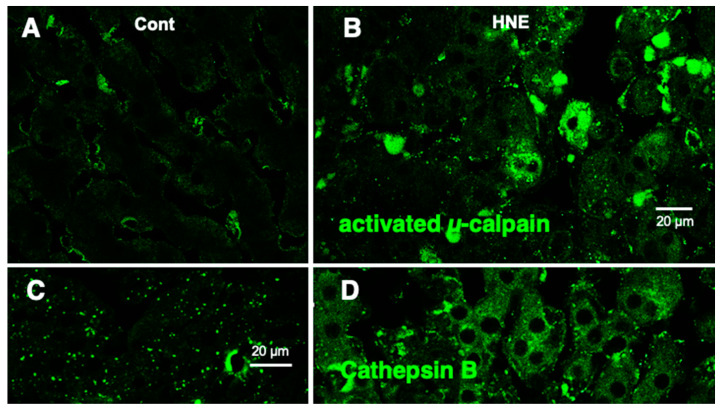
Immunofluorescence histochemical staining of activated µ-calpain (**A**,**B**) and cathepsin B (**C**,**D**). Compared to the control livers ((**A**), Cont), the livers after hydroxynonenal injections ((**B**), HNE) exhibited an increased immunoreactivity of activated µ-calpain in the hepatocytes. Concomitantly, coarse granular and diffuse cytoplasmic cathepsin B immunoreactivity was seen after the hydroxynonenal injections ((**D**), HNE), which is in marked contrast to the control ((**C**), Cont). This indicates extra-lysosomal leakage of cathepsin B, which was localized within the lysosomes prior to hydroxynonenal injections ((**C**), Cont).

Western blotting using the anti-activated μ-calpain antibody showed a significant (*p* = 0.02 in the Mann–Whitney *U* test) increase in activated μ-calpain in all four liver tissues that received hydroxynonenal injections, as reported previously [11]. This was consistent with our previous data, which showed that activation of μ-calpain was confirmed in all five patients with NASH, compared to the five non-fatty liver cases [11]. Immunofluorescence histochemical staining showed that activated μ-calpain immunoreactivity was seen as tiny granules in the hepatocytes of control monkeys, i.e., those that did not receive hydroxynonenal injections (Figure 6A), whereas, remarkably, it was significantly increased in the hepatocytes after hydroxynonenal injections (Figure 6B).

**Figure 7:**
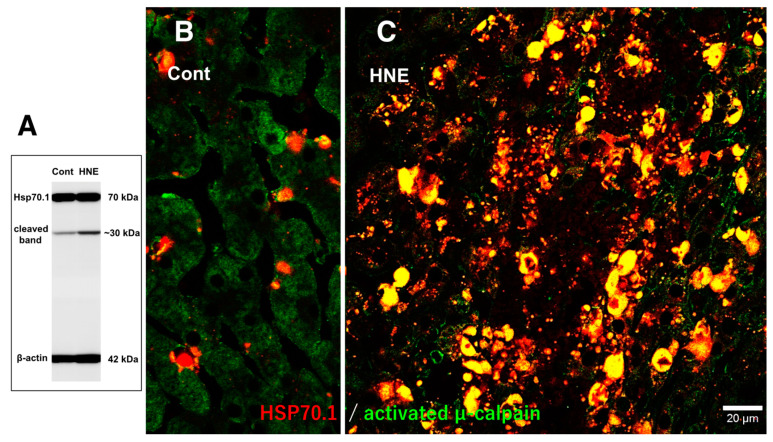
Western blotting (**A**) and immunohistochemical (**B**,**C**) data for the samples with (HNE) and without (Cont) the hydroxynonenal (HNE) injections. The Western blotting analysis indicates that the calpain-mediated cleavage of Hsp70.1 was increased after the hydroxynonenal injections (HNE), compared to the control (Cont). The immunohistochemical analysis indicates that activated µ-calpain (green) interacted with Hsp70.1 (red), especially after the hydroxynonenal injections (HNE).

In the control liver, immunofluorescence histochemical co-staining of Hsp70.1 with activated μ-calpain was only observed in the small round cells in the vicinity of the sinusoidal endothelial cells; however, most of the hepatocytes exhibited negligible immunoreactivity (Figure 7B). In contrast, after hydroxynonenal injections, the hepatocytes exhibited a remarkable increase in the merged immunoreactivity of Hsp70.1 and activated μ-calpain (Figure 7C, yellow). Interestingly, Western blotting using the same pair of monkey samples showed an increase in the Hsp70.1 cleaved band after the hydroxynonenal injections (Figure 7A). These results suggested that activated μ-calpain interacted with Hsp70.1 in the hepatocytes after the hydroxynonenal injections, which conceivably contributed to the calpain-mediated cleavage of carbonylated Hsp70.1 [20], although this is difficult to demonstrate in vivo. The presence of the Hsp70.1 protein in the 2D-DIGE analysis (Figure 4C-a) but the absence of carbonylated Hsp70.1 in the 2D-Oxyblot analysis (Figure 4C-b) were consistent with these data. Further, the immunofluorescence histochemical staining of cathepsin B revealed a low level of immunoreactivity in the control hepatocytes (Figure 6C). In contrast, the cytoplasm of the hepatocytes after the hydroxynonenal injections exhibited coarse granular staining with diffuse cytoplasmic immunoreactivity (Figure 6D). This suggests that leakage of the lysosomal contents into the cytoplasm had occurred as a result of lysosomal membrane permeabilization/rupture, presumably due to the calpain-mediated cleavage of the carbonylated Hsp70.1, as previously demonstrated in neurons after ischemia [19,20] and β-cells after hydroxynonenal injections [14]. 

**Figure 8:**
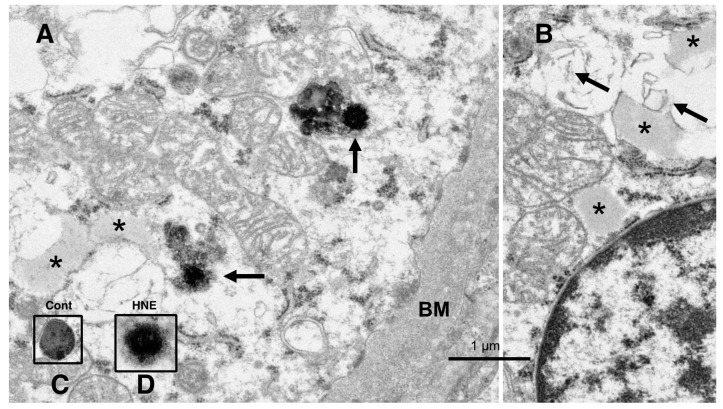
Electron microphotographs of the degenerating hepatocytes after the hydroxynonenal (HNE) injections. Compared to the membrane-bound, vivid lysosomes (**C**) in the control hepatocytes, the lysosomes of the degenerating hepatocytes after hydroxynonenal injections exhibited disintegrity of the limiting membrane ((**A**) arrows, (**D**)). In the liver after the hyfroxynonenal injections, the lysosomes often exhibited leakage of the contents or the formation of autophagolysosomes, and the normal lysosomes, such as those shown in (**C**), were extremely rare. Tiny lipid droplets ((**A**,**B**), asterisks) and membrane-derived lipid debris ((**B**), arrows) were observed in the vicinity of degenerating mitochondria and the rough ER. The basement membrane ((**A**), BM) of degenerating hepatocytes appeared thickened, which was compatible with the distinct cell boundaries as seen in the light microscopy analysis (Figure 2B). The degenerating mitochondria (**A**,**B**) exhibited mutual aggregations, which is in marked contrast to the normal mitochondria, as shown in Figure 9A and Figure 10A.

**Figure 9:**
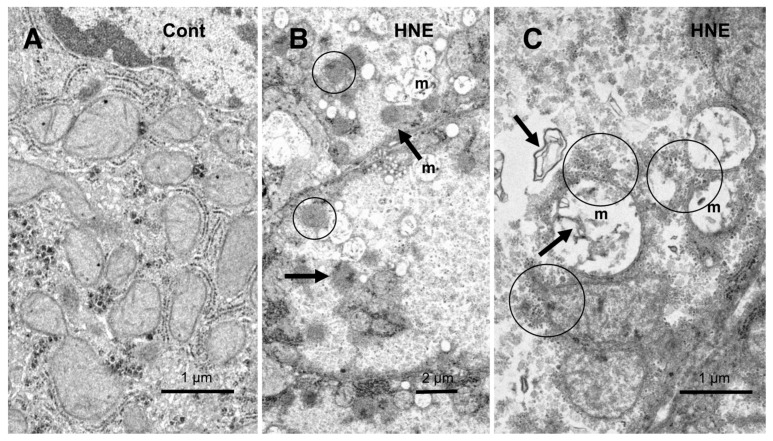
Ultrastructural changes of mitochondria and peroxisomes in the degenerating hepatocytes after the hydroxynonenal (HNE) injections (**B**,**C**), compared to the control (**A**). The cytoplasm of the control hepatocytes was filled with normal mitochondria and glycogen granules ((**A**), Cont). In contrast, most of the mitochondria ((**B**,**C**), m) in the degenerating hepatocytes exhibited a significant dissolution of the cristae with the formation of membrane debris ((**C**), HNE, arrows). The outer membrane of the degenerating mitochondria was in direct continuity with electron-dense particles ((**C**), HNE, circles). Compared to the control, abnormal peroxisomes exhibited a marked increase after the hydroxynonenal injections ((**B**), HNE, arrows). They were characterized by electron-dense deposits, an irregular configuration, and the absence of the limiting membrane ((**B**), HNE, circles).

**Figure 10:**
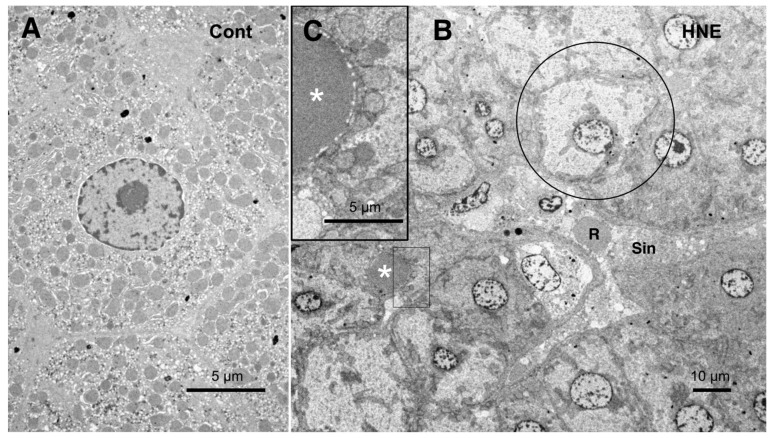
Electron microphotographs of the control hepatocytes (**A**) and degenerating hepatocytes (**B**,**C**) after the hydroxynonenal (HNE) injections. The normal hepatocytes (**A**) were characterized by numerous mitochondria. Compared to the control (**A**), the hepatocytes after the hydroxynonenal (HNE) injections (**B**) exhibited a diverse degree of degeneration after the hydroxynonenal injections; the extent of degeneration was inverse to the electron density of the cytoplasm. The electron-luscent hepatocyte ((**B**), circle) indicates a remarkable degeneration of the cytoplasm as seen in the light microscopy analysis (Figure 2B). A small lipid droplet, as observed via T-B staining (Figure 3B, arrows), formed among the degenerating hepatocytes ((**B**,**C**), white asterisks). The rectangle in (**B**) is enlarged in (**C**). R: red blood cell; Sin: sinusoidal lumen.

The electron microscopic analysis of the normal hepatocytes showed numerous mitochondria (Figures 9A and 10A) and membrane-bound, electron-dense lysosomes (Figure 8C and Figure 10A) in the hepatocytes of the control monkeys. In contrast, after hydroxynonenal injections, most of the hepatocytes showed a remarkable decrease in normal mitochondria and lysosomes (Figure 9B,C and Figure 10B), which is characteristic of degenerating hepatocytes. Furthermore, the remaining lysosomes exhibited disintegrity of the limiting membrane with an irregular configuration (Figure 8A, arrows; Figure 8D), which was associated with leakage of the lysosomal contents. 

In the hepatocytes of the control monkeys, i.e., those that did not receive hydroxynonenal injections, numerous electron-dense glycogen granules and rough ER filled the cytoplasm. In particular, the mitochondria exhibited an intact double membrane and cristae (Figure 9A). In contrast, after the hydroxynonenal injections, both the mitochondria and rough ER showed a marked decrease or intense vacuolar degeneration with loss of cristae and/or the accumulation of lipid debris (Figure 9B,C, m). Since mitochondrial function is critical to liver function, for example via the degradation of fatty acids by β-oxidation, mitochondrial degeneration and reduction were thought to be closely related to increased lipid accumulation through membrane disruption (Figure 8B and Figure 9C, arrows) [43,44]. An abundance of a finely granular substance with fuzzy material associated with disrupting mitochondrial membranes (Figure 9C, circles). Because of the impaired ATP synthesis due to damage to the mitochondria and glycogen granules, Hsp70.1 was thought to be unable to carry out autophagic removal via autophagolysosome formation because ATP is indispensable for its function. In addition, disruption of the rough ER contributed to the reduction in Hsp70.1 synthesis. Electron-dense peroxisomes devoid of a distinct limiting membrane and with an irregular configuration (Figure 9B, circles) were increased (Figure 9B, arrows) around the degenerating mitochondria (Figure 9B, m). Electron-dense glycogen granules, as seen in the normal hepatocytes (Figure 9A), disappeared. 

Instead, the cytoplasm was filled with a fine granular substance (Figure 9B,C). Both the translucent cytoplasm, as observed in the light microscopic observation (Figure 2B), and the fine granular cytoplasmic material, as observed in the electron microscopic observation (Figure 9B,C and Figure 10B, circle), indicate that the degenerating cytoplasm contained abundant fatty components, which were mostly dissolved by ethanol during the fixation for the microscopic preparation.

The low-magnification electron microscopic observation of the hepatocytes after the hydroxynonenal injections revealed the formation of an electron-dense, small lipid droplet among the degenerating hepatocytes (Figure 10B,C, asterisks). This was consistent with T-B staining findings (Figure 3B, arrows). At first glance, they were ultrastructurally similar to the red blood cells in the sinusoidal lumen, but their configuration and localization were distinct. The red blood cell was bound by a distinct membrane and was located within the sinusoidal lumen (Figure 10B, R, Sin), whereas the lipid droplet exhibited an irregular configuration and was localized among or within the degenerating hepatocytes (Figure 10B,C, asterisks). The lipid droplet was surrounded by degenerating mitochondria and peroxisomes, which were transferred to the cell periphery of degenerating hepatocytes (Figure 9B and Figure 10C). The high-magnification observation indicated the generation of a lipid-like material (Figure 10C, asterisk) in close vicinity to the degenerating mitochondrial membranes (Figure 9C, arrows, circles) and the rough ER (Figure 8B, arrows, asterisks). This membrane debris was thought to be the source of the lipid droplets, which were often seen within and in the vicinity of the degenerating hepatocytes (Figure 3B).

## 4. Discussion

Among the plethora of oxidative modifications, protein carbonylation is irreversible because of the permanent modification of the target proteins [45]. The present proteomics analyses showed a paucity of carbonylated Hsp70.1 but an increase in carbonylated BHMT. An approximately two-fold increase in carbonylated BHMT was observed (Figure 5B), although both naïve Hsp70.1 and carbonylated Hsp70.1 were downregulated, especially in the whitish-yellow portions (Figure 4B,C-a,C-b). This was consistent with a previous report, which stated that the induction of Hsp70.1 expression via stress declines significantly with age in rats and humans, presumably due to a defect in the heat shock transcription factor [46]. Hsp70.1 and BHMT disorders are critical to understanding the mechanisms of hepatocyte degeneration and steatosis after hydroxynonenal injections. Thus, two issues are separately discussed herein.

### 4.1. Hydroxynonenal-Induced Hsp70.1 Deficiency Causes Lysosomal Membrane Disintegrity and Cell Degeneration

The occurrence of NASH and its progression to cirrhosis or hepatocellular carcinoma are thought to be closely related to lipotoxicity due to free fatty acids [47]. For example, Feldstein et al. previously reported that the exposure of mouse and human hepatocytes to long-chain fatty acids induces lysosomal membrane permeabilization and the release of cathepsin B into the cytosol [48,49]. Moreover, they demonstrated that leakage of cathepsin B into the cytosol occurs in human NAFLD, whereas inhibition of cathepsin B produces protection against diet-induced fatty liver disease in mice. This is quite similar to what was reported in monkey models of transient brain ischemia, in which cathepsin release or inhibition produced neuronal death or neuroprotection, respectively [18,50]. The increase in dietary energy availability and sedentary lifestyles led to an increase in obesity, which causes an elevated level of free fatty acids in the blood. Both in vivo and in vitro, free fatty acids are known to undergo lipid peroxidation chain reactions, which, in turn, lead to the formation of highly reactive electrophilic aldehydes. As linoleic acid is the most abundant ω-6 polyunsaturated fatty acid (PUFA) in Western diets, its end products are detectable in the human blood and various organs, including the liver. Mice, which have an oxidized linoleic acid-rich diet, exhibit increased levels of hydroxynonenal in the liver [51]. The same may occur in humans as a result of high-fat diets or the daily intake of deep-fried foods cooked with ω-6 PUFAs, especially linoleic acid-rich vegetable oils [13]. 

An initial event that contributes to hepatocyte injury during NASH development is enhanced oxidative stress, which results in the short-term generation of reactive oxygen species. The latter, in turn, induces the lipid peroxidation of linoleic acids at the biomembranes and generates reactive aldehydes [52], which are a long-lasting oxidative stressor. In human NASH and in murine disease models, antibodies against reactive aldehydes show increased reactivity [53,54,55]. Although reactive aldehydes are recognized as a main cause of hepatocyte injury in NASH, the role of hydroxynonenal as a cause of chronic liver injury remains unexplored. Although there are various studies using animal models, those focusing on humans are extremely rare. For example, Bruce-Keller et al. demonstrated neuronal perikarya damage after a hydroxynonenal injection directly into the basal forebrain of rats [56]. Vigh et al. demonstrated that repeated intrathecal injections of sublethal doses of hydroxynonenal caused motor neuron loss in the spinal cord of rats [57]. In addition, intraperitoneal hydroxynonenal injections were found to exacerbate colonic inflammation through the activation of Toll-like receptor 4 signaling in mice [58]. 

Interestingly, lysosomal cell death in hydroxynonenal-induced hepatic injury was recently reported by Seike et al. [11]. Hsp70.1 is prone to calpain-mediated cleavage, especially after carbonylation by hydroxynonenal [20]. As demonstrated in hippocampal neurons after transient ischemia, calpain-mediated cleavage of carbonylated Hsp70.1 is the main cause of lysosomal membrane permeabilization/rupture. This results in the extra-lysosomal release of cathepsin enzymes, which is known as the ‘*calpain-cathepsin hypothesis*’ [18,19,20,59]. Hydroxynonenal can induce both Hsp70.1 carbonylation and calpain activation, and it facilitates the calpain-mediated cleavage of carbonylated Hsp70.1 [13]. Given that the liver has a high mitochondrial density and fast mitochondrial degradation or turnover [60], Hsp70.1 must play a crucial role in maintaining the function and health of the liver by helping in the degradation of damaged mitochondria. However, as a result of both the downregulation of Hsp70.1 in hydroxynonenal-induced liver injuries and the heterogeneity of affected lesions, immunohistochemical and immunoblot analyses could not precisely detect dynamic changes in Hsp70.1. Therefore, the proteomics analyses using 2D-DIGE and 2D-Oxyblot were indispensable in this study.

As Hsp70.1 is a stress-inducible protein that is upregulated by cell stress, decreased Hsp70.1 protein levels were correlated with the progression of NAFLD in the livers of patients with obesity [61]. Shearn et al. also found decreased expression of Hsp70 in the NASH liver in comparison with the normal human liver via the Western blotting analysis but increased carbonylation of Hsp70 in an LC-MS/MS analysis [52]. The present Western blotting analysis showed the upregulation of the Hsp70.1 protein in the pancreas (the positive control of the naïve Hsp70.1 protein) after the hydroxynonenal injections; however, the livers of the same monkeys exhibited no upregulation (Figure 4A). Instead, down-regulation of the Hsp70.1 naïve protein was confirmed by both Western blotting (Figure 4B-left) and 2D-DIGE analyses (Figure 4C-a). In addition, both SDS-PAGE (Figure 4B-right) and 2D-Oxyblot (Figure 4C-b) failed to detect carbonylated Hsp70.1. This is in marked contrast to the hippocampus after transient ischemia, which exhibited more than a 10-fold increase in carbonylated Hsp70.1 on day 5 postischemia (Figure 4C-c) [35] but was consistent with the results of NAFLD [61] and NASH [52]. However, the immunohistochemical analysis showed an intense cross-immunoreactivity of activated μ-calpain with Hsp70.1 (Figure 7C), which indicates that activated μ-calpain is involved in the cleavage of carbonylated Hsp70.1 (Figure 7A). This is very similar to what was observed for ischemic hippocampal neurons.

There should be certain differences in the response of Hsp70.1 to acute insults, such as transient brain ischemia, and chronic insults, such as consecutive hydroxynonenal injections. We speculate that a 24 week exposure to hydroxynonenal was such a severe long-term insult to the monkey liver that carbonylated Hsp70.1 was continuously and steadily cleaved by activated μ-calpain (Figure 7A) at the time of each hydroxynonenal injection. Presumably, this lasted 24 weeks, i.e., until Hsp70.1 deficiency was complete at the point of tissue resection. Because Hsp70.1 is a stress-inducible protein that is characterized by a short life span and fast turnover, severe disruption of the rough ER and mitochondria (Figure 8, Figure 9 and Figure 10) presumably caused both synthesis impairment and Hsp70.1 dysfunction (Figure 4C-a) in the hepatocytes after the repeated hydroxynonenal injections. Protein synthesis impairment combined with the increased cleavage of Hsp70.1 (Figure 4A and Figure 7A) conceivably contributed to the Hsp70.1 deficiency (Figure 4C-a).

Fatty acids are mainly oxidized in mitochondria through the subsequent reactions of β-oxidation. Acetyl-CoA is produced from fatty acids, and this enters the TCA cycle to generate energy by completely oxidizing it to CO_2_ [62]. Archer et al. recently found that Hsp70.1 is important in maintaining mitochondrial integrity and fatty acid oxidation in the liver; therefore, it is capable of both preserving hepatic homeostasis and preventing lipid storage [63]. In this study, along with mitochondrial degeneration, a remarkable proliferation of abnormal peroxisomes was observed in the degenerating hepatocytes. Peroxisomes are single membrane-bounded organelles that are well-known for their involvement in lipid metabolism and redox balance [64,65,66]. Peroxisomes contain several oxidases involved in the production of H_2_O_2_ and catalase, which is involved in the decomposition of H_2_O_2_ to oxygen and water [67,68]. To absorb nutrients that the cell has acquired, peroxisomes digest long-chain fatty acids and break them down into smaller molecules via β-oxidation. The representative byproducts of β-oxidation are H_2_O_2_ and hydroxyradicals (OH•). Ultrastructurally, the proliferating peroxisomes did not exhibit a limiting membrane with an irregular configuration (Figure 9B), as shown in the pancreatic Langerhans β-cells in the same monkeys after the hydroxynonenal injections [14]. Accordingly, we speculate that abnormal peroxisomes, which were found in the degenerating hepatocytes, are related to the sustained generation of reactive oxygen species and β-oxidation disorder. 

### 4.2. BHMT Carbonylation and Cleavage Cause Phosphatidylcholine Decrease and Hepatic Steatosis

There are three major dietary sources of methyl groups in ‘one carbon metabolism’: methionine, betaine, and choline. Methionine is an essential sulfur-containing gluconeogenic amino acid that is required for normal development and cell growth. Using ATP as a co-substrate, it is converted to *S*-adenosylmethionine by the enzyme methionine adenosyltransferase. In addition, the methyl group of *S*-adenosylmethionine is transferred to a large variety of substrates, such as DNA, RNA, proteins, phosphatidylethanolamine, glycine, and guanidinoacetate for cell homeostasis. These reactions are called ‘transmethylation reactions’ and are catalyzed by specific methyltransferases. Methionine is also regenerated from homocysteine via methionine synthase, using vitamin B_12_ as a cofactor and vitamin B_9_ as a methyl donor. In addition, BHMT catalyzes methionine synthesis from homocysteine using betaine, which is synthesized from choline by the enzyme choline oxidase as a methyl donor [69]. Choline is a nutrient obtained through both dietary intake and endogenous synthesis. It is either oxidized to form betaine by the enzyme choline dehydrogenase or used to generate phosphatidylcholine (the CDP–choline pathway). Choline deficiency leads to a deficiency of phosphatidylcholine. Choline deficiency also perturbs protein kinase C signaling, which results in altered cell proliferation signals [70]. Therefore, both humans and rats with a choline deficiency develop both fatty liver [70,71] and hepatocyte death and liver damage [72]. In addition, the product of BHMT, i.e., methionine, is required for the synthesis of phosphatidylcholine from phosphatidylethanol-amine via the phosphatidylethanolamine methyltransferase pathway (the PEMT pathway). These two pathways generate phosphatidylcholine in the liver [26]. Studies of phosphatidylcholine anabolism in mice indicate that 70% of the hepatic synthesis of phosphatidylcholine is derived from the CDP–choline pathway, requiring dietary choline, while 30% is derived from the PEMT pathway [23]. Since BHMT influences hepatic lipid accumulation, the deletion of the BHMT protein perturbs choline metabolism and alters the phosphatidylcholine concentration by disturbing the above two pathways. 

The carbonylation of BHMT was previously reported using mass spectrometry in a rat model of alcoholic steatosis, which was characterized by the accumulation of fat in the liver 3 and 6 weeks after ethanol exposure [36]. Furthermore, mice with the gene deletion encoding BHMT (*Bhmt*^−/−^) developed a fatty liver and showed an elevation of ALT at 5 weeks of age. The *Bhmt*^−/−^ mice liver exhibited a 21-fold increase in betaine and a ~27% decrease in phosphatidylcholine concentrations, possibly due to the increased use of choline to form betaine. Consequently, a six-fold increase was observed in hepatic triglyceride concentrations compared to the wild-type mice (*Bhmt*^+/+^), which was due to a decrease in the secretion of VLDL [30]. Since even a ~27% decrease in phosphatidylcholine can cause a six-fold increase in hepatic triglyceride concentrations, it is probable that a slight decrease in phosphatidylcholine concentration, as observed in this study, can contribute to lipid depositions in the monkey liver to some extent. Phosphatidylcholine is a membrane constituent, and in the liver, it is crucial for the efflux of VLDL [27,73,74]. It is required for the assembly/secretion of lipoproteins in the liver [75] and for solubilizing cholesterol in bile [76]. Accordingly, the link between choline/phosphatidylcholine deficiency and hepatic steatosis was well recognized more than half a century ago [77]. The present experimental paradigm confirmed the tendency of phosphatidylcholine to decrease in two of the three samples studied. This was compatible with the decrease in the BHMT naïve protein (Figure 5A), the increase in BHMT cleavage (Figure 5A), and the increase in the carbonylation of BHMT (Figure 5B). These data together suggest that calpain-mediated cleavage of carbonylated BHMT occurred in response to hydroxynonenal. 

Alterations in choline and phosphatidylcholine metabolism may have the impact of predisposing the subject to fatty liver. Concerning the mechanism of poor choline and phosphatidylcholine availability in the liver, there are three explanations that are widely accepted at present: (1) low dietary intake; (2) low estrogen status; and (3) genetic polymorphisms. All three of these affect the de novo phosphatidylcholine synthesis pathway [26]. Although exposure to alcohol [36] or vegetable oils [13] appears to be distinct with regard to cellular toxicity, the alcohol metabolite ‘acetaldehyde’ and the lipid peroxidation product ‘hydroxynonenal’ actually have the same active group (–CHO). Regardless of the designation as ‘–nal’ or ‘–aldehyde’, both hydroxynonenal and acetaldehyde can cause protein carbonylation. One should note that the consumption of linoleic acid-rich vegetable oils and the consumption of alcohol have exactly the same adverse effect in terms of generating –nal (–aldehyde) in the body. The concept of ‘acquired BHMT disorder due to hydroxynonenal-mediated carbonylation followed by cleavage’ has never been reported before. Importantly, we should keep in mind that phosphatidylcholine deficiency due to BHMT disorder may occur more drastically in older people with an increased hydroxynonenal serum concentration due to the reduction in detoxification enzymes [78]. In particular, in those with an aldehyde dehydrogenase 2 (ALDH2) gene mutation [79], we speculate that the long-term intake of vegetable oils causes hepatic steatosis via the accumulation of hydroxynonenal in a similar manner to the BHMT disorder related to acetaldehyde-induced ALDH2 deficiency in rats after ethanol exposure [36]. 

In this study, we utilized synthetic hydroxynonenal to study the adverse effects of the vegetable oil-peroxidation product ‘hydroxynonenal’ using an experimental monkey model. Because the reproduction of the serum levels observed in human patients with Alzheimer’s disease via the administration of synthetic hydroxynonenal induced severe liver injury in all four monkeys studied, it is likely that hydroxynonenal resulting from vegetable oil peroxidation, if generated in excess, especially in elderly people and those with an ALDH2 deficiency [78,79], will produce similar adverse effects in humans. Exogenous hydroxynonenal is generated within minutes of deep frying linoleic acids, i.e., it is in oils and/or foods that we cook daily, while endogenous hydroxynonenal is generated in the biomembranes as a result of circumferential and/or intrinsic oxidative stress. Accordingly, hydroxynonenal-induced BHMT disorder should be considered an explanation for phosphatidylcholine deficiency and hepatic steatosis. Because either exogenous or endogenous hydroxynonenal can cause lysosomal membrane destabilization via Hsp70.1 deficiency [80] and the resulting hepatocyte degeneration/death with steatosis, individuals should consider reducing their daily intake of ω-6 PUFA-rich vegetable oils as they are detrimental for the liver.

## 5. Summary

Lipotoxicity involves very complex cellular mechanisms in which excess adiposity results in cell degeneration/death. However, the exact molecular cascade involved in lipotoxicity in the liver has not been elucidated in detail until now. The ‘*calpain-cathepsin hypothesis*’ explains the involvement of the lipid peroxidation product ‘hydroxynonenal’ in ischemic neuronal death, i.e., calpain-mediated cleavage of carbonylated Hsp70 induces lysosomal membrane permeabilization/rupture in the brain. Here, we studied whether a similar molecular cascade works in the liver. Intriguingly, we found that reductions in phosphatidylcholine, increases in lipid depositions, lysosomal membrane disintegrity, dissolution of mitochondria and rough ER, and hepatocyte degeneration/death all occurred via Hsp70.1 and BHMT disorders in the monkey liver after consecutive hydroxynonenal injections. Accordingly, it was concluded from the present study that targeting ‘hydroxynonenal’ would be a viable strategy to prevent chronic liver diseases. In those whose diets include the daily intake of linoleic acid-rich vegetable oils and/or who have high-fat diets, Hsp70.1 deficiency and acquired BHMT disorder should be recognized as a potential cause of hepatic injury and steatosis.

## Abbreviations

ALDH2	aldehyde dehydrogenase 2
AST	aspartate aminotransferase
ALT	alanine aminotransferase
γ-GTP	γ-glutamyl transferase
BHMT	betaine-homocysteine *S*-methyltransferase
DNP	2,4-dinitrophenylhydrazone
DNPH	2,4-dinitrophenylhydrazine
Hsp70.1	heat-shock protein 70.1
MALDI-TOF/TOF MS	matrix-assisted laser desorption ionization time-of-flight tandem mass spectrometry
NAFLD	nonalcoholic fatty liver disease
NASH	nonalcoholic steatohepatitis
PEMT pathway	phosphatidylethanolamine methyltransferase pathway
PUFA	polyunsaturated fatty acid
2D-DIGE	two-dimensional differential in-gel electrophoretic
VLDL	very low-density lipoprotein
